# Effects of smoking on cognition and BDNF levels in a male Chinese population: relationship with BDNF Val66Met polymorphism

**DOI:** 10.1038/s41598-018-36419-8

**Published:** 2019-01-18

**Authors:** Haisen Xia, Xiangdong Du, Guangzhong Yin, Yingyang Zhang, Xiaosi Li, Junyi Cai, Xingbing Huang, Yuping Ning, Jair C. Soares, Fengchun Wu, Xiang Yang Zhang

**Affiliations:** 1 Hefei Fourth People’s Hospital, Anhui Mental Health Center, Hefei, China; 2 Suzhou Psychiatric Hospital, The Affiliated Guangji Hospital of Soochow University, Jiangsu, China; 3 The Affiliated Brain Hospital of Guangzhou Medical University (Guangzhou Huiai Hospital), Guangzhou, China; 4 Department of Psychiatry and Behavioral Sciences, The University of Texas Health Science Center at Houston, Houston, Texas USA; 5 Institute of Psychology, Chinese Academy of Sciences, Beijing, China

## Abstract

Recent studies demonstrate that brain-derived neurotrophic factor (BDNF) might be associated with nicotine addiction, and circulating BDNF is a biomarker of memory and general cognitive function. Moreover, studies suggest that a functional polymorphism of the *BDNF* Val66Met may mediate hippocampal-dependent cognitive functions. We aimed to explore the relationships between smoking, cognitive performance and BDNF in a normal Chinese Han population. We recruited 628 male healthy subjects, inducing 322 smokers and 306 nonsmokers, and genotyped them the *BDNF* Val66Met polymorphism. Of these, we assessed 114 smokers and 98 nonsmokers on the repeatable battery for the assessment of neuropsychological status (RBANS), and 103 smokers and 89 nonsmokers on serum BDNF levels. Smokers scored lower than the nonsmokers on RBANS total score (p = 0.002), immediate memory (p = 0.003) and delayed memory (p = 0.021). BDNF levels among the smokers who were Val allele carriers were correlated with the degree of cognitive impairments, especially attention, as well as with the carbon monoxide concentrations. Our findings suggest that smoking is associated with cognitive impairment in a male Chinese Han population. The association between higher BDNF levels and cognitive impairment, mainly attention in smokers appears to be dependent on the *BDNF Val66Met* polymorphism.

## Introduction

Despite the significant health risks resulting from tobacco use, the prevalence of smoking worldwide remains high and approximately 20% of the world population still smokes tobacco^1,2^. In China, over 60% of adult men are current smokers^3,4^. Substantial evidence demonstrates that nicotine has high addiction liability as well as cognition-enhancing effects^5^. Individuals may self-medicate with nicotine to enhance cognitive and attentional processes^6^, which may partially account for the high prevalence of cigarette smoking among individuals with cognitive disorders such as schizophrenia, attention deficit/hyperactivity disorder, Alzheimer’s and Parkinson’s diseases, and age related cognitive decline^7–9^. A recent meta-analysis also showed that nicotine could enhance aspects of cognitive function, including motor abilities, attention and memory^9^. Moreover, this nicotine-induced enhancement of cognitive function has been observed both in nicotine-deprived smokers, in non-deprived smokers, and in non-smokers^5,10^. However, several studies suggest that smoking has an adverse effect on cognition^11–14^. Some studies show that long-term smokers perform more poorly on tests of cognition later in life than never-smokers, and ex-smokers perform better than those who continue to smoke^14^. Longitudinal studies have suggested a causal relationship between chronic smoking into late life and an increased risk of cognitive decline and dementia^11–13^. Thus, the smoking-cognition relationship warrants further investigation in a large sample of individuals.

Brain-derived neurotrophic factor (BDNF), a member of the neurotrophin family, is widely expressed in the adult mammalian brain, the highest levels being found in the hippocampus, followed by the cerebral cortex^15^. BDNF plays an important role in the regulation of neuronal survival, differentiation, growth, and apoptosis^16^. Studies have shown that BDNF can modulate activity-dependent synaptic plasticity underlying learning and memory in the hippocampus^17,18^. More specifically, BDNF appears to play an important role in both early long term potentiation (LTP) and late phase LTP in hippocampal neurons^19^, a cellular model of learning and memory^17^. Previous studies indicate that circulating BDNF may be a biomarker of memory and general cognitive function in healthy adults^20,21^. Several studies have shown that BDNF serum levels are significantly decreased in individuals with cognitive decline-related diseases, such as mild cognitive impairment^21^, Alzheimer’s disease^22^, and Huntington’s disease^23^. Interestingly, our own recent study showed that decreased BDNF levels were associated with cognitive impairment, especially immediate memory in schizophrenia^24,25^.

A single-nucleotide polymorphism (SNP) in the *BDNF* gene (rs6265) produced a valine (Val)-to-methionine (Met) substitution in the proBDNF protein at codon 66 (Val66Met). This SNP altered the intracellular trafficking and activity dependent secretion of mature BDNF and affected hippocampal function^18,26–29^. Consistent with this, both normal controls and schizophrenia patients with Met alleles have significant deficiencies in episodic memory, suggesting that Met carriers show a poorer cognition in both schizophrenia and healthy subjects^18,30,31^. These findings support a role for the *BDNF* Val66Met polymorphism in normal hippocampal dependent memory function in healthy controls as well as in individuals with schizophrenia^18^.

Preclincal data show that acute nicotine administration decreases whereas chronic nicotine, but not cocaine or morphine, increases BDNF mRNA levels in the hippocampus^32^. Interestingly, genome-wide linkage scans indicate that the region of chromosome 11p13 where the *BDNF* gene is located likely harbors susceptibility genes for nicotine dependence specifically^33^. Moreover, Beuten *et al*. provide evidence of an association between allelic variants of *BDNF* and nicotine dependence in male European-American smokers^34^. A recent study demonstrated that the frequencies of both the Met/Met genotype and Met allele were significantly increased in current and in former smokers when compared to never smokers among Germans^35^, suggesting that carriers of the *BDNF* Met allele have an increased risk for smoking. While subsequent association studies did not corroborate these findings^24,36^, recent studies have linked this *BDNF* polymorphism with smoking initiation^37^, smoking cessation^38^ and the age of smoking initiation^24^. Moreover, a lower plasma concentration of BDNF was reported in the smoking group^39^. However, a recent study indicated that smoking was associated with increased plasma BDNF levels in depressed patients^40^. The most recent study by Jamal *et al*. showed that smokers with and without nicotine dependence had higher levels of serum BDNF than former and never-smokers. Moreover, total number of smoking years was a significant predictor of serum BDNF. However, there was no association of *BDNF* Val66Met, nor an interaction of this polymorphism and smoking status, with serum BDNF^41^. Interestingly, they further found that nicotine-dependent smokers had more severe depressive symptoms than non-dependent smokers, former and never-smokers. Moreover, in Val66Val carriers, nicotine-dependent smokers had more severe symptoms of depression and anxiety than the other three groups, suggesting that *BDNF* Val66Met genetic variants may be crucial for the worse behavioral outcome of nicotine^42^. Our recent study showed that smoking was associated with cognitive decline, but not with BDNF levels in a normal population. Concomitant alcohol use did not worsen the cognitive decline and had no effect on BDNF levels. However, smoking severity was positively associated with BDNF levels^43^. Taken together, these findings suggest that smoking may affect the peripheral BDNF levels, and BDNF system may be involved in the etiology of nicotine dependence.

Given the established association between smoking and cognitive performance and the important role of BDNF in cognition and in nicotine dependence, it would be of interest to explore the relationships between smoking, cognitive performance and BDNF in a healthy population. We hypothesized that smoking may enhance some aspects of cognitive function in a Chinese Han population. The underlying mechanisms by which smoking may enhance cognitive performance might be associated with higher BDNF serum levels. Furthermore, Met carriers might show a poorer cognitive performance in smokers or non-smokers, or both. In addition, the relationship between serum BDNF levels and cognitive function may be dependent on the *BDNF* Val66Met polymorphism in smokers. Because smoking is substantially more common among Chinese men than in women with normal population^44^, as well as gender differences in smoking behaviors^45^, we included only male subjects in the present study.

## Results

Table 1 shows demographic and clinical characteristics of the smokers and nonsmokers. The mean age, education, marriage, and body mass index (BMI) did not differ between the two groups (all p > 0.05).

**Table 1:** Characteristics of smokers and non-smokers.

	Smokers (N = 322)	Nonsmokers (N = 306)		F	P
Age (years)	46.6 ± 11.8		45.8 ± 13.3	0.46	>0.05
Education (years)	9.1 ± 3.5		9.2 ± 3.6	0.21	>0.05
BMI (Kg/m^2^)	25.4 ± 6.1		24.9 ± 5.8	0.89	>0.05
BDNF (ng/ml)^a^	11.6 ± 2.3		11.5 ± 3.0	0.23	>0.05
Allele frequency (%)					
Val	339 (52.6%)		304 (49.7%)	1.11	>0.05
Met	305 (47.4%)		308 (50.3%)		
Genotype frequency (%)				2.26	>0.05
Val/Val	81 (25.1%)		74 (24.2%)		
Met/Val	177 (55.0%)		156 (51.0%)		
Met/Met	64 (19.9%)		76 (24.8%)		

Note: BMI = body mass index; BDNF = brain-derived neurotrophic factor.

^a^BDNF serum level data were available from 103 smokers and 89 nonsmokers.

Distributions of the *BDNF* genotypes were consistent with HWE in both smokers and nonsmokers (both p > 0.05). We found no significant differences in *BDNF* genotype and allele distributions between smokers and nonsmokers (x^2^ = 2.26, df = 2, p > 0.05; and x^2^ = 1.11, df = 1, p > 0.05, respectively) (Table 1).

### Genotype effects on cognitive functioning between smokers and nonsmokers

RBANS data were available from 114 smokers and 98 nonsmokers. Table 2 shows RBANS total and index scores, and the effects of the *BDNF* Val66Met polymorphism on the RBANS total and index scores.

**Table 2:** Comparisons of RBANS total and index scores by smoking status and BDNF Val66Met genotype.

Cognitive Index	Smokers			Nonsmokers			Genotype, F (*P* value)	(*P* value)
	Val/Val (n = 23)	Val/Met (n = 71)	Met/Met (n = 20)	Val/Val (n = 19)	Val/Met (n = 58)	Met/Met (n = 21)		
Immediate Memory	72.0 ± 13.9	71.2 ± 16.9	76.8 ± 13.4	82.0 ± 14.4	79.7 ± 18.4	74.8 ± 16.1	0.14 (0.87)	1.81 (0.17)
Attention	83.9 ± 15.4	90.0 ± 13.8	88.2 ± 20.2	95.5 ± 17.9	92.5 ± 23.	90.4 ± 20.4	0.23 (0.8)	1.03 (0.36)
Language	93.3 ± 12.1	96.0 ± 8.9	91.5 ± 9.2	95.7 ± 11.7	99.8 ± 13.9	93.0 ± 13.8	**4.25 (0.016)** ^a^	0.16 (0.85)
Visuospatial/Constructional	79.7 ± 18.3	80.2 ± 13.0	82.7 ± 18.3	84.4 ± 10.4	83.1 ± 17.6	79.5 ± 18.0	0.04 (0.96)	0.77 (0.47)
Delayed Memory	82.3 ± 13.8	84.6 ± 14.5	86.9 ± 14.7	94.1 ± 7.7	88.7 ± 16.7	86.2 ± 14.4	0.20 (0.82)	1.92 (0.15)
Total	77.0 ± 12.5	79.0 ± 10.6	80.7 ± 12.9	87.3 ± 11.2	85.5 ± 18.1	80.5 ± 14.6	0.23 (0.80)	1.50 (0.23)

Note: ^a^There were significant genotype effects on Language index (F = 4.25, p = 0.016).

Smokers performed significantly worse than nonsmokers on the RBANS total score (t = 3.09, df = 210, p = 0.002), immediate memory (t = 3.02, df = 210, p = 0.003) and delayed memory (t = 2.32, df = 210, p = 0.021). After the Bonferroni correction (α = 0.05/6 = 0.0083), only the significant difference in the RBANS total score and immediate memory remained significant. After adjusting for age, education and BMI, these results still remained significant for RBANS total score (F_4,204_ = 7.95, p = 0.005), and for immediate memory (F_4,204_ = 9.60, p = 0.002). However, there were no significant differences in other cognitive measures between smokers and nonsmokers (all p > 0.05).

As shown in Table 2, the three genotypes significantly differed on the language index (F = 4.25, p < 0.02), and using the Bonferroni test to control for the two potential comparisons only the Val/Met vs. Met/Met difference was significant (p = 0.027). There were no significant genotype effects for other indices or RBANS total scores.

### Genotype effects on serum BDNF levels between smokers and nonsmokers

Serum BDNF levels were available from 103 smokers and 89 nonsmokers. These 192 subjects with BDNF level test overlapped with those subjects who had the RBANS test. Serum BDNF levels were normally distributed in both smokers and nonsmokers (Kolmogorov–Smirnov one-sample test: both p > 0.05). There was not a significant main effect of smoking status on BDNF levels (11.6 ± 2.3 ng/ml for smokers versus 11.5 ± 3.0 ng/ml for nonsmokers, p > 0.05). Furthermore, there was no main effect of genotype on serum BDNF levels (p > 0.05) nor genotype × smoking status effect (p > 0.05).

### Differential effects of BDNF genotype on the relationships between BDNF serum levels and cognitive functioning

Since some cognitive domain impairments existed in smokers, we explored whether the cognitive impairments may be related to serum BDNF levels and whether such relationships differed between genotype groupings in smokers. We first tested the overall effect of the BDNF levels on all five cognitive domain scores in a multivariate ANCOVA test. BDNF was the independent variable, and the 5 cognitive domains and RBANS total scores were entered simultaneously as dependent variables. Covariates in the multivariate ANCOVA test were age, education and BMI. A BDNF level-by-genotype interaction term was also entered in the model. The results showed a trend toward significant overall main effect of BDNF on neurocognition (F = 1.96, p = 0.08). The BDNF level-by-genotype interaction term was statistically significant (F = 2.98, p = 0.012), which indicated that the relationships between BDNF levels and cognitive domain scores differed between genotype groupings. Further, post hoc univariate analyses found significant BDNF effects on attention (F = 6.92, p = 0.01), and a trend toward significant effect on RBANS total score (F = 2.89, p = 0.09). Moreover, we also found a significant BDNF level-by genotype interaction effect on language score (F = 3.91, p = 0.024). However, these significances did not pass the Bonferroni corrections (all p > 0.05).

In the smoking group, partial Pearson correlations were significant between BDNF levels and attention (r = −0.28, df = 98, p = 0.014) and non-significant between BDNF and RBANS total score (r = −0.21, df = 98, p = 0.06). Interestingly, BDNF levels were significantly associated with CO levels (r = 0.38, df = 96, p < 0.01), and non-significantly with FTND (r = 0.24, df = 95, p = 0.06). However, only the significant association between BDNF and CO levels passed the Bonferroni corrections (p < 0.05).

Table 3 shows Pearson partial correlation coefficients for the relationships between BDNF levels and cognitive domain scores within each genotype group in the smoking group. Among the Val/Met genotype, BDNF levels were significantly associated with worsening attention performance (r = −0.34, df = 65, p = 0.019). Further, this association was observed especially in Val allele (Val/Met + Val/Val) carriers (r = −0.38, df = 86, p = 0.002). In addition, among the Val/Met genotypes, BDNF was significantly associated with CO concentrations (Val/Met: r = 0.33, df = 65, p = 0.022). Further, this association was observed especially in Val allele carriers (r = 0.38, df = 86, p = 0.006). However, these significant associations did not pass the Bonferroni test (all p > 0.05).

**Table 3:** Relationships between BDNF levels and cognitive domain scores and carbon monoxide concentration across BDNF Val66Met genotype groupings in smokers.

Cognitive	Val/Val (n = 21)		Val/Met (n = 65)		Met/Met (n = 17)	
	Pearson		Pearson		Pearson	
Domains	Partial *r*	p	Partial *r*	p	Partial r	p
Immediate memory	−0.06	0.84	−0.07	0.66	−0.07	0.80
Attention	−0.49	0.06	−0.34	**0.019**	−0.04	0.90
Language	−0.22	0.45	−0.02	0.90	−0.48	0.07
Visuospatial/Constructional	−0.37	0.20	−0.17	0.24	0.10	0.72
Delayed Memory	0.13	0.66	−0.08	0.58	−0.38	0.16
Total	−0.42	0.13	−0.18	0.23	−0.18	0.53
CO (ppm)	0.44	0.09	0.33	**0.022**	0.16	0.59

Note: CO = carbon monoxide; ppm = parts per million.

In nonsmoking groups, no significant correlation was found between BDNF level and any RBANS index or total scores. Moreover, no significant correlation between BDNF level and any RBANS index and total scores was observed when being grouped by *BDNF* genotype (all p > 0.05), indicating no significant effects of BDNF level, *BDNF* genotype and their interaction on cognitive performance in the nonsmoking group.

## Discussion

To our best knowledge, this is the first study to investigate the effect of smoking on BDNF and cognition as well as their relationship in a normal Chinese population. The main findings of the present study were that, (1) smokers had significantly lower cognitive scores on the RBANS total score, immediate memory and delayed memory; (2) there was no significant difference in BDNF serum levels and *BDNF* Val66Met genotype between smokers and nonsmokers; (3) there was a significant negative association between serum BDNF levels and attention only in the smoking group. Moreover, this association was only observed in Val allele carriers; (4) BDNF level was found to be significantly associated with CO levels in the smoking group. Moreover, this association was only observed in Val allele carriers.

The finding of lower cognitive performance in smokers is concordant with numerous studies evaluating cognitive function in smokers among the normal population^11–14^, suggesting that chronic smoking may increase the cognitive decline in a normal population. However, some previous studies suggest that nicotine could enhance aspects of cognitive function, including motor abilities, attention and memory^9^, especially in some diseases that have cognitive deficits, such as schizophrenia, attention deficit/hyperactivity disorder, Alzheimer’s and Parkinson’s diseases, and age related cognitive decline^6–8^. Nicotine’s cognition-enhancing effects have been attributed to enhancing DA signaling in mesolimbic and mesocortical DA pathways^5^, an up-regulation of nicotinic acetylcholine receptors (nAChRs), or possibly protection from the neurotoxicity induced by free radicals^8^. However, nicotine also can produce neuronal toxicity, and chronic cigarette smoking potently increases free radical production and antioxidant depletion^46^. Moreover, the other 4700 components of tobacco smoke include a variety of carcinogens as well as other toxic compounds such as carbon monoxide, heavy metals and cyanide^47^, and could account for some of the cognitive, intellectual, and behavioral impairments in the non-demented elderly^48^. Interestingly, a previous study showed that nicotine’s effects may be dose dependent, and low dose nicotine may be an antioxidant and be neuroprotective, whereas high dose nicotine may induce neurotoxicity through oxidative stress and cellular injury^49^. Taken together, we hypothesize that smoking or nicotine use, especially at low dose and acute administration, may improve cognition in those patients with established cognitive deficits, but long-term use at high doses may damage cognition in healthy controls, suggesting that regular smoking or nicotine use may have a different effect on cognitive function in healthy controls compared to patients with cognitive deficits.

We found no association between the *BDNF* Val66Met polymorphism and smoking among male Chinese, which is consistent with two recent studies in a healthy Caucasian population^36,41,42^, but not with two other studies in European-Caucasians^34,35^. A possible reason for the inconsistencies in genetic association studies is related to ethnic background. For example, the allele frequency distribution of Val66Met has varied significantly between Chinese and Caucasian subjects. The Met allele frequency is around 50% in Chinese consistent with the 48.8% Met allele frequency found in our current study, but it is only around 20% in Caucasian subjects^50^. Thus, interethnic differences in the genotype frequencies of the *BDNF* Val66Met polymorphism may play an important role in accounting for the inconsistent results across the different populations. Other factors may include different sample sizes, the methodologies for measuring smoking, and ethnic stratification effects^10^. Thus, the association of the *BDNF* gene polymorphism and smoking behavior deservers further investigation.

Interestingly, we found a significant negative association between serum BDNF levels and attention only in the smoking group, and this association was only observed in Val allele carriers. Thus, BDNF was implicated in cognitive function only in smokers. Furthermore, the *BDNF* Val66Met polymorphism affected the correlation between BDNF and different aspects of cognitive function only in smokers, not in nonsmokers. The BDNF serum level displayed a negative association with attention, but the association was opposite to our expected direction. Studies have shown that BDNF plays a critical role in activity dependent neuroplasticity underlying learning and memory in the hippocampus^17,18^. Recent studies have shown that BDNF levels are significantly decreased in individuals with cognitive decline-related diseases, such as mild cognitive impairment, Alzheimer disease, and Huntington’s disease^20–23^. Moreover, higher BDNF levels are associated with better neuropsychological test performance in these diseases^21^. However, it is noteworthy that in our present study, smokers had some cognitive deficits, but not lower BDNF levels. Hence, it is possible that smoking-induced cognitive deficits (maybe through free radical mechanisms) lead to an increase in BDNF levels in order to reverse smoking’s harmful effects, and prevent further damage. Hence, increases in BDNF levels may be compensatory processes to attain homeostasis in the cognition-BDNF system, which might lead to higher BDNF levels in smokers. However, in our current study, we found no significant difference in BDNF levels between smokers and nonsmokers, suggesting that smokers had already failed to have sufficient BDNF. Moreover, comparatively high BDNF being associated with more cognitive impairment in smokers needs to be viewed in the context of no overall differences in BDNF in these smokers compared to nonsmokers. In addition, our findings of the significant association between BDNF levels and CO concentrations and the trend toward significant association between BDNF and FTND total score seem to support this hypothesis that greater nicotine dependence or smoking severity elevates BDNF in smokers.

Furthermore, *BDNF* Val66Met genotype groupings had a significant impact on the relationship between BDNF levels and cognitive impairments in smokers. The correlations between BDNF levels and cognition especially attention was only observed for Val allele carriers, but not for the Met/Met genotype. These divergent serum BDNF- cognitive function relationships across *BDNF* genotypes in smokers may also provide a clue about the unusual finding of higher BDNF levels with more cognitive impairment. A direct effect of the *BDNF* Val66Met polymorphism itself may have contributed toward the present findings. This polymorphism has been found to be associated with altered intracellular trafficking of pro-BDNF and secretion of the mature peptide^18,27,28^, suggesting that the *BDNF* Val66Met variant is a kind of functional polymorphism. Moreover, the Met66 allele has been associated with impaired depolarization-dependent secretion and intracellular trafficking of BDNF protein in the hippocampus^18^. Thus, the functional Val66Met polymorphism of the *BDNF* gene may exert its effect on the cognitive performance of smokers by affecting BDNF expression and protein production. Our failure to observe an association between BDNF levels and cognition in the smoking group with the Met/Met genotype could be due to lack of enough BDNF to counteract the smoking-induced cognitive impairment. However, while the functional effect of the Met66 allele on trafficking of the protein makes this polymorphism a strong candidate for direct involvement in attention function in smokers, we cannot exclude linkage disequilibrium with another polymorphism nearby as an explanation for this association.

Further, it is worthy of mentioning that Met allele carriers have demonstrated poorer episodic memory than their respective Val-homozygous counterparts in healthy controls or in patients with schizophrenia^18,31^. Moreover, Met allele carriers have lower hippocampal function and have reduced hippocampal and prefrontal gray matter^30^, brain regions known to participate in memory. However, the current study showed association with other aspect of neuropsychological functioning, such as attention and language, but not in the memory domain. We could not provide the reasonable explanations for the mechanisms by which the *BDNF* Val66Met variant affects attention, but not memory due to our cross-sectional design in our current study. It is possible that this discrepancy might be related to the study design differences.

Several limitations of this study should be noted. First, this is a cross-sectional study design and cannot show direct causality, although there were many significant associations or correlations in our study. That means, they are not etiology. For example, the BDNF correlation with cognition was only presented in smokers, but not in non-smokers, suggesting that BDNF changes and cognition changes are all downstream effects of smoking, which are pathological changes. Second, both smokers and nonsmokers had a relatively limited sample size, especially when being divided into subgroups by the *BDNF* genotype. This could lead to false positive or negative results due to the lack of statistical power and selection of samples. Moreover, after the Bonferroni correction, none of the associations remained significant, suggesting a small effect. Hence, a replication study would be needed with a larger sample size. Third, due to the shortcoming of the design for this study and the limits of the experimental and clinical conditions, we only had about 200 subjects who carried out the cognitive test and were on serum BDNF level test, although all the 628 subjects were genotyped with *BDNF-* Val66Met polymorphism. This might lead to bias in the statistical analysis due to the imbalance in the number of the subjects who took cognitive test, BDNF serum assay and *BDNF-* Val66Met polymorphism genotyping differentially. Fourth, we only measured the BDNF serum levels following an overnight fast, but not during and after smoking in smokers. Previous studies have shown that smoking influenced the BDNF serum levels^39,40^. We speculate that BDNF serum levels might change at the different time points of smoking, which warrants further investigation. Besides smoking, exercise level, alcohol and food may affect the BDNF serum levels, which had not been collected in this study and will be remedied in the future investigation. Fifth, our sample was limited to only males and can not be applied to females. It would be interesting to evaluate whether our findings in the present study could generalize to female subjects. Sixth, we focused on the Val66Met polymorphism, because it is functional and previously associated with cognitive performance. However, in our current study, we found that the serum BDNF levels were not related to val66met genotype. We speculated that other genetic variants within *BDNF* may influence subsequent BDNF expression or activity, suggesting more extensive genotyping to identify haplotype blocks of greater association with cognition. Although we did not find the effects of Val66Met polymorphism on serum BDNF levels, we did find a significant negative association between serum BDNF levels and attention only in Val allele carriers in the smoking group, suggesting the important role of this polymorphism in adjusting the relationship of cognition and serum BDNF levels. Seventh, several cognitive performance tests were used, but there was only a correlation with the attention index and BDNF in the smoker group. We could not give a reasonable explanation why only one domain of cognitive performance was associated with BDNF in the smoker group due to the nature of our cross sectional design. Moreover, the significance is weak, and the positive results were based on a comparatively small sample size. Eighth, we measured BDNF in serum, not in cerebral spinal fluid (CSF). It is still uncertain whether peripheral BDNF levels reflect similar changes in the central nervous system (CNS).

In summary, we have provided new evidence to support that smoking is associated with significant cognitive impairment in a male Han Chinese population, especially on immediate and delayed memory, and total cognitive performance. Although we did not find any associations of BNDF levels or the *BDNF* Val66Met polymorphism with smoking, we found a significant negative association between serum BDNF levels and attention and a positive association between BDNF levels and CO concentrations in the smoking group. Moreover, these associations in smokers appear to be dependent on the presence of *BDNF* Val66Met polymorphism, with these associations observed only in Val allele carries. However, these findings still need to be regarded as preliminary because of the relatively small sample size and the cross-sectional design in this study. Future large controlled prospective studies would help clarify the interrelationships between smoking, cognitive functioning and BDNF.

## Methods

### Subjects

Six hundred and twenty-eight healthy unrelated male volunteers (aged 22–70 years, mean age: 46.3 ± 12.7 years) were recruited from the Haidian district in Beijing. The participants were interviewed by trained investigators, using a detailed questionnaire including general information, socio-demographic characteristics, current and previous smoking behavior, and medical and psychological conditions. All subjects met the following inclusion criteria: 1) age 20–70 years, Han Chinese; 2) no DSM-IV Axis I psychiatric diagnosis by a research psychiatrist using the basis of the Structured Clinical Interview for DSM-IV (SCID); 3) being able to provide the signed informed consent for participating in the study and to complete the cognitive test.

A complete medical history, physical examination and lab test were obtained from all subjects. Those subjects with physical diseases were excluded. All of the subjects were excluded from alcohol or illegal drug abuse/dependence by self-report.

Subjects meeting one or more of the following criteria were excluded: (1) documented disease of the central nervous system that could interfere with the cognitive assessments including, but not limited to epilepsy, stroke, tumor, Parkinson’s disease, Huntington’s disease, seizure disorder, history of brain trauma resulting in significant impairment; (2) acute, unstable and/or significant and untreated medical illness (e.g., infection, diabetes, hypertensions); (3) chronic infection; (5) a clinically significant electrocardiogram (ECG) abnormality in the opinion of the investigator; (6) use of any medication; (7) history of severe allergy or hypersensitivity; (8) dependence on alcohol or illicit drugs.

The Institutional Review Board (IRB) at Beijing HuiLongGuan hospital approved this study and each subject gave written informed consent for participating after the study had been fully explained. In addition, all methods were performed in accordance with Declaration of Helsinki promulgated by the National Institute of Health.

In order to maintain consistency in the use of various terms while gathering data on smoking behavior, the US Centers for Disease Control and Prevention have developed and updated the following definitions^2^: *Never smokers* – Adults who have never smoked a cigarette or who have smoked fewer than 100 cigarettes in their entire lifetime. *Former Smokers* – Adults who have smoked at least 100 cigarettes in their lifetime, but they currently do not smoke. *Nonsmokers* – Adults who currently do not smoke cigarettes, including both former smokers and never smokers. *Current Smokers* – Adults who have smoked 100 cigarettes in their lifetime and currently smoke cigarettes every day (daily) or some days (nondaily). Former smokers were excluded from the present study.

In addition, the Chinese translation of the standardized Fagerstrom Test for Nicotine Dependence (FTND) was employed to measure the degree of nicotine dependence (ND)^51^. Additional visits were requested for subjects with missing or ambiguous data. Exclusion criteria included a history of -Diagnostic and Statistical Manual of Mental Disorders—Fourth Edition | (DSM-IV) psychiatric disorder, alcohol abuse, and other drug abuse. Demographic data are summarized in Table 1.

### Cognition Measures

We assessed cognitive functioning using the repeatable battery for the assessment of neuropsychological status (RBANS, Form A)^52^ from 212 subjects on the same day as the blood samples were taken. The RBANS is comprised of 12 subtests that are used to calculate 5 age-adjusted index scores and a total score. Test indices are Immediate Memory (comprised of List Learning and Story Memory tasks); Visuospatial/Constructional (comprised of Figure Copy and Line Orientation tasks); Language (comprised of Picture Naming and Semantic Fluency tasks); Attention (comprised of Digit Span and Coding tasks); and Delayed Memory (comprised of List Recall, Story Recall, Figure Recall, and List Recognition tasks). The RBANS was previously translated into Chinese by our group and its clinical validity and test–retest reliability were established among controls^53^.

### Measurement of carbon monoxide

Measurement of carbon monoxide (CO) concentrations in exhaled air was performed using BreathICO (Vitalograph Buckingham, UK). Three hundred and thirty-six participants (143 smokers and 193 non-smokers) were instructed to hold their breath for 15 seconds and then exhale into a disposable tube attached to the BreathICO. Breath CO concentrations were expressed in parts per million (ppm).

### Serum BDNF measurement

Blood samples were collected between 06:00 and 08:00 following an overnight fast. We measured fasting serum BDNF levels from 192 subjects by sandwich ELISA using a commercially available kit as described in our previous report^54^. All samples were assayed by a research assistant blind to the clinical situation. Inter- and intra-assay variation coefficients were 7 and 5%, respectively.

### Genotyping

The genotypes of the *BDNF* Val66Met polymorphisms from all subjects (n = 628) were identified as reported in our previous study^43^. A research assistant who was blinded to the clinical status genotyped every subject twice for accuracy of genotyping^55–58^.

### Statistical Analysis

Deviation from Hardy–Weinberg equilibrium was calculated by chi-square (χ^2^) goodness-of-fit test. The *BDNF* Val66Met allele and genotype frequencies were compared between smokers and nonsmokers using x^2^ tests. Group differences were compared using Student’s two-sample *t*-test or one-way analysis of variance (ANOVA) for continuous variables and chi-squared for categorical variables. Correlation between variables was studied using Pearson product moment correlations.

We tested associations between the *BDNF* Val66Met polymorphism and the cognitive measures or serum BDNF levels using a general factorial design in PASW statistical software version 18.0 (SPSS Inc, Chicago, Illinois). For the main models, the *BDNF* Val66Met genotype and smoking status (smokers vs nonsmokers) were entered as fixed effects. Scores for each cognitive domain and the total scores of RBANS were entered as the dependent variables, with age, education and body mass index (BMI) included as covariates as appropriate. In each model, the main effect of smoking status group, the main effect of genotype and smoking group × genotype interaction were tested. The smoking group × genotype interaction term in the model detects the differential effects that alleles might have on cognitive scores between smoking status groups. Similarly, the main effect of the *BDNF* genotype on serum BDNF levels was also analyzed using analyses of covariance (ANCOVA). We applied Bonferroni corrections to adjust for multiple testing. Lastly, we performed exploratory analyses to examine whether the relationships between BDNF serum levels and cognitive function were different across *BDNF* Val66Met genotype groups. Partial Pearson’s product moment correlation coefficients were used to assess associations of cognitive performance with serum BDNF levels while adjusting for various potentially confounding demographic variables of age, education and BMI. Statistical significance was defined as p < 0.05.
